# A Phase I Dose-Escalation Study of Lenalidomide in Combination with Gemcitabine in Patients with Advanced Pancreatic Cancer

**DOI:** 10.1371/journal.pone.0121197

**Published:** 2015-04-02

**Authors:** Gustav J. Ullenhag, Eva Rossmann, Maria Liljefors

**Affiliations:** 1 Department of Radiology, Oncology and Radiation Science, Section of Oncology, Uppsala University, Uppsala, Sweden; 2 Department of Oncology, Uppsala University Hospital, Entrance 78, 751 85 Uppsala, Sweden; 3 Department of Oncology and Pathology (Radiumhemmet), Cancer Centre Karolinska, Karolinska Institutet, Karolinska University Hospital Solna, Stockholm, Sweden; Indiana University School of Medicine, UNITED STATES

## Abstract

**Purpose:**

Lenalidomide have both immunomodulatory and anti-angiogenic properties which could confer anti-cancer effects. The aim of this study was to assess the feasibility of combining lenalidomide with the standard treatment gemcitabine in pancreatic cancer patients with advanced disease.

**Patients and Methods:**

Eligible patients had locally advanced or metastatic adenocarcinoma of the pancreas. Patients received lenalidomide days 1–21 orally and gemcitabine 1000 mg/m2 intravenously (days 1, 8 and 15), each 28 day cycle. Three cohorts of lenalidomide were examined (Cohort I = 15 mg, Cohort II = 20 mg and Cohort III = 25 mg daily). The maximum tolerated dose (MTD) of lenalidomide given in combination with gemcitabine was defined as the highest dose level at which no more than one out of four (25%) subjects experiences a dose-limiting toxicity (DLT). Patients should also be able to receive daily low molecular weight heparin (LMWH) (e.g. dalteparin 5000 IU s.c. daily) as a prophylactic anticoagulant for venous thromboembolic events (VTEs). Twelve patients (n = 4, n = 3 and n = 5 in cohort I, II and III, respectively) were enrolled in this study.

**Results:**

Median duration of treatment was 11 weeks (range 1–66), and median number of treatment cycles were three (range 1–14). The only DLT was a cardiac failure grade 3 in cohort III. Frequent treatment-related adverse events (AEs) (all grades) included neutropenia, leucopenia and fatigue (83% each, but there was no febrile neutropenia); thrombocytopenia (75%); dermatological toxicity (75%); diarrhea and nausea (42% each); and neuropathy (42%).

**Discussion:**

This phase I study demonstrates the feasibility of the combination of lenalidomide and gemcitabine as first-line treatment in patients with advanced pancreatic cancer. The tolerability profile demonstrated in the dose escalation schedule of lenalidomide suggests the dosing of lenalidomide to be 25 mg daily on days 1–21 with standard dosing of gemcitabine and merits further evaluation in a phase II trial.

**Trial Registration:**

ClinicalTrials.gov NCT01547260

## Introduction

Pancreatic cancer is characterised by aggressive growth, treatment resistance and poor prognosis [1]. The majority of patients presents with advanced, inoperable disease. The five-year survival rate is less than 5% [2]. Even those patients who are candidates for surgical resection experience a poor prognosis with a five-year survival rate of only 8–20% [3, 4].

For patients with advanced disease, gemcitabine is standard treatment which may be combined with 5-FU. The median survival is 5.6 months [5]. Several studies of gemcitabine combined with other cytotoxic chemotherapy agents have failed to improve survival compared with gemcitabine alone [6]. Targeting the vascular endothelial growth factor (VEGF) receptor in pancreatic carcinomas using bevacizumab [7] or axitinib [8] in combination with gemcitabine did not improve survival. However, FOLFIRINOX has been shown to improve overall survival in patients with metastatic disease when compared to gemcitabine, but with added toxicity [9]. In addition, targeting the epidermal growth factor receptor (EGFR) (erlotinib) has shown to improve survival in combination with gemcitabine. The modest improvement in survival was statistically significant in patients with metastatic, but not in locally advanced disease [10]. Furthermore, overall survival was extended with two months in median with the addition of nab-paclitaxel to gemcitabine in pancreatic cancer patients with metastatic disease (MPACT trial) [11]. Thus, irrespective of treatment regimens, survival of pancreatic cancer patients remains poor and new therapeutic strategies are urgently needed.

Lenalidomide (Celgene Corporation, Summit, NJ) is a thalidomide analogue that was approved by the U.S. Food and Drug Administration (FDA) and by the European Medicine Agency (EMA) for relapsed or refractory multiple myeloma (MM) [12, 13]. Although the antitumour mechanism of action of lenalidomide is not fully understood, a number of mechanisms have been postulated, involving both immunomodulatory and anti-angiogenetic properties [14]. Lenalidomide has been demonstrated to possess anti-angiogenic activity through inhibition of bFGF, VEGF and by TNF-alpha induced endothelial cell migration [15]. Lenalidomide also has immunomodulatory and anti-inflammatory effects by augmenting natural killer (NK)—cell cytotoxicity [16], by regulating T-cell co-stimulation [16–18] and by altering cytokine production [19], which could confer antitumor activity [20].

In solid tumors, safety and potential clinical efficacy of lenalidomide has been observed in patients with advanced disease who have previously received multi-modality treatment receiving lenalidomide administered as single [21–24] or as combination therapy [25]. The combination of pomalidomide, the 3rd generation of Immunomodulatory drugs (IMiDs), with gemcitabine, was safe in most patients as first-line treatment for metastatic pancreatic cancer [26]. In addition, treatment with lenalidomide combined with gemcitabine has been explored in metastatic pancreatic cancer patients [26, 27].

Beside the cytotoxic activity of gemcitabine, accumulating evidence has indicated that the product promote specific anticancer immune responses that contribute to the therapeutic effects. Gemcitabine may augment immune responses in several ways; By activating T cells [28] increasing the amount of DC:s [29] increasing the amounts of antigens loaded onto antigen-presenting cells (APC) [30], down-regulating T-regulatory cells [31, 32], and IL-6 [33]. Administration of gemcitabine can make the tumor cells more susceptible to T-cell mediated destruction by inducing up-regulation of death receptors [34] and has shown to enhance immune responses against cancer vaccines [28]. Those data supports that lenalidomide and gemcitabine should be of major interest to explore for combination therapy. The primary objective of this study was to determine the maximum tolerated dose (MTD) of lenalidomide in combination with gemcitabine as first line treatment for patients with advanced pancreatic cancer. There is no previous dose escalation study with this combination in pancreatic cancer patients.

## Patients and Methods

The protocol for this trial and supporting TREND checklist are available as supporting information; see S1 Checklist and S1 Protocol.

To our knowledge, all ongoing and related trials for this drug/intervention are registered.

### Patient population

Eligible patients had histologically or cytologically confirmed unresectable, locally advanced, or metastatic adenocarcinoma of the pancreas. No prior chemotherapy for metastatic disease or locally advanced disease was allowed. Participants may have been previously treated with gemcitabine, fluorouracil, or capecitabine in the adjuvant setting. Patients should also be able to receive daily low molecular weight heparin (LMWH) (e.g. dalteparin 5000 IU subcutaneously daily) as prophylactic anticoagulant. Female subjects of childbearing potential should agree to use effective contraception without interruption, 4 weeks before starting study drug, throughout the study and for 4 weeks after end of study drug therapy. A negative urine pregnancy test in women of child-bearing potential before the start of treatment was required.

Male subjects must agree to use condoms throughout study drug therapy and for one week after cessation of study therapy. Other eligibility criteria included: age >18 years, Eastern Cooperative Oncology Group (ECOG) performance status of 0 or 1, life expectancy > 12 weeks, adequate bone marrow function (defined as absolute neutrophil count (ANC) >1.5 x 10^9^/L, platelet count >100 x 10^9^/L), adequate renal and hepatic function (defined as serum creatinine <2,0 mg/dL (< 177 μmol/L) and total bilirubin < 3 x the institutional upper limit of normal (ULN), AST/SGOT and ALT/SGPT <3 x ULN or < 5 x ULN for patients with liver metastases.

Exclusion criteria included prior systemic therapy for adenocarcinoma of the pancreas

(except in the adjuvant setting, see above), use of any other experimental therapies within 28 days prior to Cycle 1 Day 1, a history of or active deep vein thrombosis (DVT) or pulmonary embolism (PE) that were not managed on a stable dose of appropriate anticoagulant, known brain metastases, prior history of malignancy within 5 years (except basal or squamous cell carcinoma or carcinoma in situ of the cervix or breast, localized prostate cancer with PSA < 1.0 mg/dL) or pregnant or lactating females. An independent data and safety monitoring board (DSMB) monitored data throughout the study with respect to the occurrence of secondary primary malignancies (SPMs).

Patients were treated according to the Declaration of Helsinki’s ethical principles for medical research involving human subjects. The trial was performed according to GCP guidelines and was approved by the Regional Ethical Review Board for each Institution on the 6^th^ of October 2009 (Regionala etikprövningsnämnden i Uppsala and Regionala etikprövningsnämnden i Stockholm) and by the Medical Products Agency in Uppsala, Sweden. The study was registered at ClinicalTrials.gov only after inclusion began since registration was not a routine procedure in Sweden in 2009. All patients provided an informed written consent prior to study entry.

### Study design and treatment assessment

This was a dual-agent, two-centre, open-label phase I study conducted at the Karolinska University Hospital Solna, Sweden and Uppsala University Hospital, Uppsala, Sweden. The opportunity to participate included referred patients. The primary objective of this dose-escalation part of the study (phase I) was to determine the maximum tolerated dose (MTD) and safety of lenalidomide in combination with gemcitabine in patients with untreated advanced pancreatic cancer.

Lenalidomide was administered orally once daily for 21 days of a 28 day cycle. The prescribed dose of lenalidomide was given as a single dose each morning. Gemcitabine was administered by a nurse at a fix dose of 1000 mg/m^2^ intravenously over 30 minutes, on days 1, 8 and 15 every 28 day. All patients received prophylactic low molecular weight heparin (LMWH) (dalteparin, Pfizer Inc. New York, USA) (5000 IU s.c. once daily) during lenalidomide treatment. If platelet count was < 50 x 10^9^/L, anticoagulant therapy was withheld until recovery to platelet count to > 50 x 10^9^/L. All patients were on treatment until disease progression, unacceptable toxicity or consent withdrawal.

The dose-escalation scheme of lenalidomide was as follows; cohort I: capsule lenalidomide 15 mg once daily (dose-level 1), cohort II: capsule lenalidomide 20 mg once daily (dose-level 2) and cohort III: capsule lenalidomide 25 mg once daily (dose-level 3) (Table 1). The lenalidomide dose in cohort III was the highest planned dose per protocol. This dose was chosen based on results from previous phase I clinical trials data in patients with MM [35] and solid malignancies, including pancreatic carcinoma, using lenalidomide as a single-agent [22–24], or combined with other cytotoxic drugs, but with the addition of G-CSF treatment [25].

**Table 1:** Dose escalation and reduction schedule.

**Dose level**	**Cohort**	**Lenalidomide dose**	**Gemcitabine dose**
		**(mg/day)**	**(mg/m** ^2^ **)**
		**Days 1–21 of each**	**Days 1, 8 and 15 of each**
		**28-day cycle**	**28-day cycle**
**-2**		5	500
**-1**		10	750
**1**	I	15	1000
**2**	II	20	1000
**3**	III	25	1000

The dose of lenalidomide was escalated in sequential cohorts of three patients each. Dose-escalation continued if none of three patients experienced a dose-limiting toxicity (DLT). A cohort was expanded to four patients if one of the first three patients had a DLT during the first treatment cycle. There are several strategies for dose escalation when combining two drugs in phase I trials [36, 37]. Since gemcitabine is an established treatment for these patients, only lenalidomide was dose escalated and we used a modified 3:3 design in order to rapidly establish MTD. Enrollment to dose-cohorts level 2 and level 3 was withheld until the last enrolled patient in the previous dose-cohort had reached the end of cycle 1, i.e. at least 28 days of cycle 1. The maximum tolerated dose (MTD) of lenalidomide was defined as the highest dose level at which no more than one out of four (25%) patients experiences a DLT. If two or more out of four patients within the same cohort encounter DLT, MTD was exceeded and the lower dose level was considered to be MTD. Adverse events (AEs) were defined by Common Terminology Criteria for Adverse Events (CTCAE) version 3.0. DLTs were evaluated during the first treatment cycle (28 days) and defined as follows: a) inability to deliver all scheduled doses during cycle 1 due to an unexpected drug-related toxicity (if gemcitabine-related toxicity was an expected toxicity, it was not considered a DLT), and/or b) inability to deliver the intended doses of lenalidomide in cycle 1 due to drug-related toxicity as outlined below: any grade 3 or 4 non-hematological toxicity lasting for ≥ 14 days, febrile neutropenia, any grade 4 neutropenia lasting for ≥ 7 days, grade 4 thrombocytopenia or grade 4 liver enzyme toxicity. Grade 3 or 4 venous thromboembolic events were not considered to DLT as long as anticoagulant therapy could be administered. Criteria for safety evaluation were completion of the first treatment cycle or early discontinuation owing to DLT.

Patients who did not finish the first cycle due to AEs other than DLTs were replaced.

Events were classified as Not suspected (the relationship of the AE to study drug made a causal relationship unlikely or remote, or other medications, therapeutic interventions, or underlying conditions provided sufficient explanation for the observed event) or Suspected (the relationship of the AE to study drug made a causal relationship possible, and other medications, therapeutic interventions, or underlying conditions did not provide sufficient explanation for the observed event). All AEs were recorded by the Investigator(s) during the period between start of the first cycle (Day 1) until 30 days following the last dose of study drug administration.

### Dose modification and interruption

Dose-modifications were not permitted for lenalidomide during cycle 1 in any dose cohort, unless DLT occured. If the patient experienced drug-related toxicity assessed as related to gemcitabine during cycle 1, the gemcitabine dose was modified to dose level -1 at day 8, see Table 1. If AE not restored to grade ≤ 2 within 7 days, dose reduction to dose level -2 of gemcitabine was advised at day 15. In subsequent cycles, dose-modifications and/or interruptions for both lenalidomide and gemcitabine were prescribed (Table 1) in patients who developed hematologic or non-hematologic toxicities related to the treatment.

The dose of lenalidomide and gemcitabine in the subsequent cycle was based on toxicity and dose-reduction noted in the previous cycle. Additionally, before initiating a new treatment cycle, the following conditions had to be met; neutrophil count ≥ 1.0 x 10^9^/L, platelet count ≥ 75 x 10^9^/L, lenalidomide-related allergic reaction/hypersensitivity or sinus bradycardia/ other cardiac arrhythmia that may have noted had to be resolved to ≤ grade 1 severity. Other lenalidomide/gemcitabine related adverse events that may had been noted had to be resolved to ≤ grade 2 severity.

### Assessments

Prior to treatment (baseline-visit), patients were evaluated by a complete medical history and physical examination including assessment of ECOG performance status, complete blood count (including differential and platelet counts), serum chemistry (including electrolytes and liver function tests), coagulation tests (international normalized ratio (INR), and activated partial thromboplastin time (APT), thyroid stimulating hormone (TSH) levels and serum tumour marker (CA 19–9), urine pregnancy test (in women of child-bearing potential) and electrocardiogram (ECG) as well as an computer tomography (CT) scan of the chest and abdomen.

During treatment, patients were examined every 28th day. On day 1 of each cycle, patients underwent interval medical history and physical examination, assessment of ECOG performance status, evaluation of adverse events and concomitant medications. Complete blood counts were repeated days 1, 4–5, 8, 11–12, 15, 21–23 during cycle 1, days 1, 8, 15, 21–23 during cycle 2 and days 1, 8 and 15 of the subsequent cycles. Serum chemistry including CA19-9 was repeated on day 1 of each cycle. TSH levels were analysed every second month. At the completion of every other cycle, disease assessments were performed by CT scans. All patients were monitored for secondary primary malignancies. At treatment discontinuation, patients underwent evaluations as at the baseline-visit. Safety assessments were also done approximately 28 days post last dose of study drug.

### Treatment compliance

At all times, when the study drug was dispensed, research center personnel reviewed the instructions, printed on the packaged together with the patients. The patients were asked to keep a diary. Research personnel counted and recorded the number of used and unused study drug capsules at each visit and reconciled with the patient diary.

### Concomitant therapy

Patients received supportive care, including transfusions of blood and blood products, antibiotics, and antiemetics when appropriate. The use of granulocyte colony-stimulating factor (G-CSF) was permitted after cycle 1. Concomitant use of chemotherapy, radiation, or other investigational agents was not allowed while the patients received study drug.

### Statistical analysis

Data from all patients who received one or more doses of drug were included in the analyses. Descriptive statistics were used to analyse and present the data.

## Results

An electronic case report form (eCRF), PheedIt (SAS Institute) was used to record the results.

### Clinical characteristics

Thirteen patients from two clinical sites were enrolled from the 14^th^ January 2010 to the 20^th^ May 2011. One patient did not receive lenalidomide during cycle one, due to drug exchange mistake (protocol violation). The patient was taken out of the study immediately. As the patient did not receive the allocated intervention, he is not included in the analysis. An additional patient was screened for the study and signed the informed consent in December 2009 but she never started treatment since her condition deteriorated rapidly. The first follow up was on the 12^th^ October 2010 and the last follow up was on the 10^th^ April 2014. Baseline demographic and clinical characteristics of the patients are shown in Table 2. Median time from initial diagnosis of advanced disease to start of treatment with lenalidomide and gemcitabine was 9.5 weeks (range 4–208 weeks).

**Table 2:** Patients baseline characteristics and number of patients per cohort.

**Patient no.**	**Sex/age (years)**	**ECOG Performance status**	**Site of metastasis at inclusion**	**Previous treatment** *	**Time from diagnosis of advanced disease to start of study (weeks)**	**Cohort**	**Dose lenalidomide (mg/day)**
**111**	M/64	0	Liver	None	53	I	15
**112**	F/63	0	Liver	Surgery	10	I	15
**113**	M/64	1	Liver	None	4	I	15
**114**	M/79	0	Liver	Surgery	208	I	15
**121**	F/69	1	Liver	None	9	II	20
**122**	F/68	1	Lungs	None	6	II	20
**123**	M/69	0	Peritoneum	None	7	II	20
**131**	M/44	1	Peritoneum	Surgery	9	III	25
**133**	M/65	0	Nodes	None	4	III	25
**134**	M/68	0	Nodes	None	10	III	25
**135**	M/62	0	Liver	None	10	III	25
**136**	F/55	0	Liver	Surgery and chemotherapy#	51	III	25

*Surgery = Pancreaticoduodenectomy.

#Adjuvant chemotherapy with gemcitabine and capecitabine for 6 months

### DLT and MTD

No DLTs were observed in cohorts I (lenalidomide dose 15mg/day) or II (lenalidomide dose 20mg/day). One patient in cohort I (patient no. 113) developed urticaria grade 3 and erythema grade 2 at day 6 in cycle 1. The lenalidomide administration was temporarily held. It was not considered a DLT but the patient was replaced to have an adequate number of patients to determine MTD. One other patient in cohort III (patient no. 131) developed abdominal pain grade 3, vomiting grade 2 and grade 3 rash at day 7 in cycle 1. The nausea/vomiting and rash persisted for 7 days while the grade 3 abdominal pain continued after the hold of both study drugs. The symptoms were considered to be related to the underlying disease and therapy was permanently withheld. The patient was non-evaluable for DLT and an additional patient was enrolled in the cohort.

The only DLT was a cardiac failure grade 3 in cohort III (patient no 135). The patient had no sign of preexisting cardiac disease at the baseline visit. The patient complained of moderate fatigue and dyspnea on exertion but not at rest, at day 26 of cycle 1. Chest-X-ray showed enlargement of the heart compared to baseline and apical vascular redistribution, compatible with left-sided heart failure. Echocardiography showed a marginal dilatation of the left atrium but the ejection fraction was within normal limits. There were no changes in the ECG including QTs interval. Routinely performed blood tests indicating heart disease were normal. By lowering the dose of lenalidomide in the subsequent cycles, the patient was on therapy for further nine cycles without any clinical symptoms or signs of cardiac failure.

After the DLT of a transient cardiac failure at the 25-mg dose level, a second additional patient was enrolled in cohort III. Dose-escalation was stopped at the dose of 25 mg of lenalidomide in combination with gemcitabine 1000 mg/m^2^, the highest planned dose level.

### Treatment exposures, delays and dose reductions

A total of 64 cycles of lenalidomide in combination with gemcitabine were administered. The numbers of cycles and treatment duration are shown in Table 3. The median number of treatment cycles per patient was three (range 1–14). Median treatment duration was 9 weeks (range 7–23 weeks), 9 weeks (range 8–11 weeks), and 44 weeks (range 1–66 weeks) for patients in cohort I, cohort II and cohort III, respectively. For all patients, median treatment duration was 11 weeks (range 1–66 weeks).

**Table 3:** Treatment exposures, number of treatment cycles and median delivered dose of the study drugs.

**Pat no.**	**Cohort**	**Dose lenalidomide (mg/day) Days 1–21 of each 28-day cycle**	**No. of treatment cycles**	**Dose (%)** ** **Cycle 1**		**Median Dose (%)** *** **Cycle 2 and beyond**		**Treatment duration (weeks)**
				Lenalidomide	Gemcitabine	Lenalidomide	Gemcitabine	
**111**	I	15	2	100	85	100	75	7
**112**	I	15	6	100	100	85	80	23
**113**	I	15	2	30	100	67	75	7
**114**	I	15	3	100	85	79	84	11
**121**	II	20	3	100	100	50	67	8
**122**	II	20	3	100	100	100	100	11
**123**	II	20	3	100	85	67	50	9
**131**	III	25	1	30	70	NA*	NA	1
**133**	III	25	14	100	100	80	78	66
**134**	III	25	13	100	100	40	51	56
**135**	III	25	10	100	100	55	90	44
**136**	III	25	4	100	100	100	100	16

*NA = not applicable

** Administered dose / planned dose according to the protocol per cohort (%)

*** Median administered dose / planned dose according to the protocol per patient per cohort (%)

**Fig 1:**
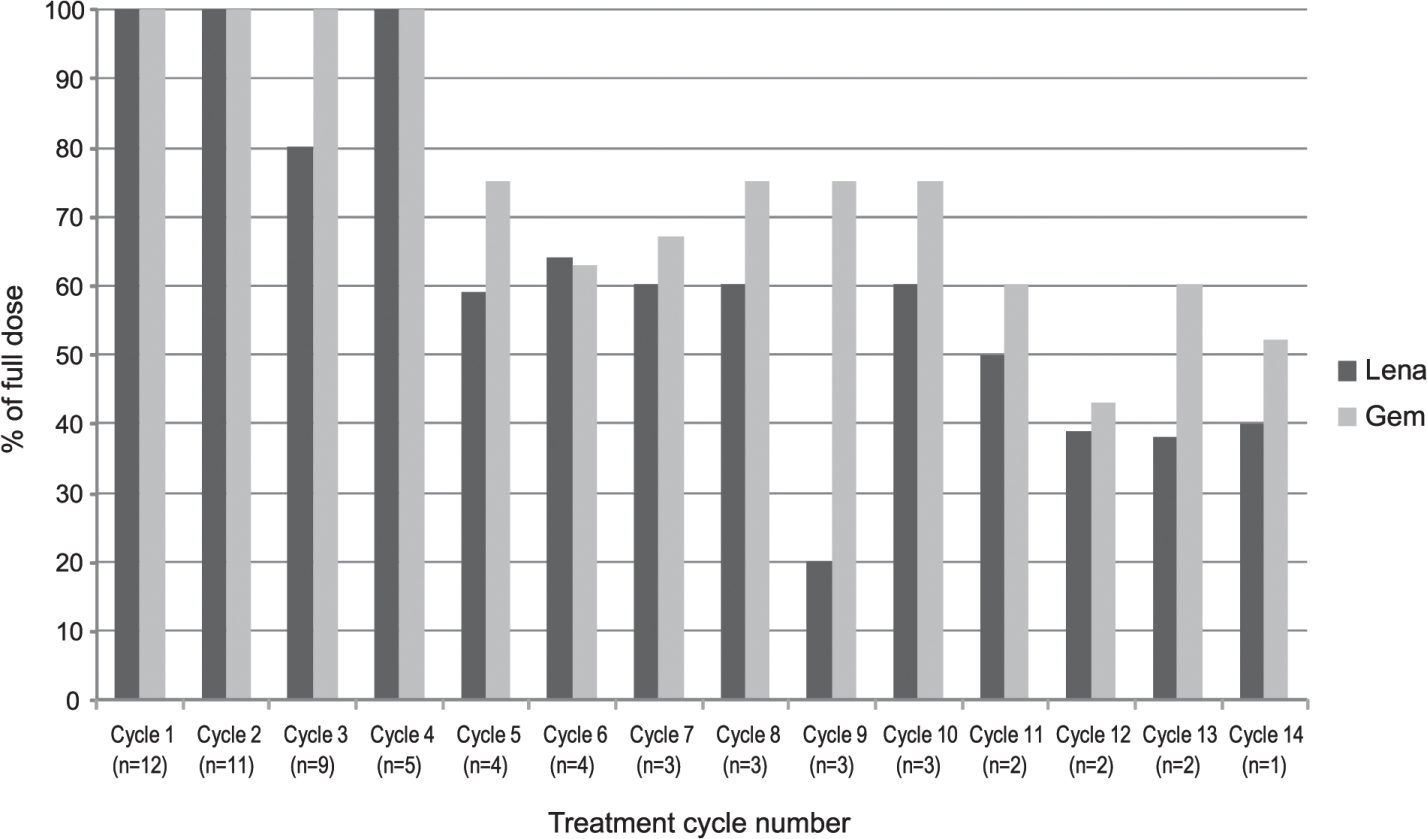
Median delivered doses (% of full doses) for lenalidomide (black bars) and gemcitabine (grey bars), respectively, in treatment cycles 1–14 for all patients on study. For an individual patient, the dose of lenalidomide per cycle was calculated by the following formula: the actual administered dose (mg) multiplied by the number of actual treatment days (n) *divided* by the planned administered dose (mg) multiplied by the planned treatment days (n) according to the protocol per cohort.

In treatment cycle no. 1, the median delivered dose of the intended dose was 100% for both lenalidomide (range 30–100%) and gemcitabine (range 70–100%) (Table 3). From treatment cycle no. 2, the corresponding values were 79% (range 40–100%) for lenalidomide and 78% (range 50–100) for gemcitabine. Fig 1 shows the median delivered doses (in % of full doses) for lenalidomide and gemcitabine, respectively, in treatment cycles 1–14 for all patients.

For an individual patient, the dose of gemcitabine per cycle was calculated by the following formula: the actual administered dose (1000 mg/m^2^ x any dose reduction in % if appropriate) multiplied by the number of actual treatment occasions (n) *divided* by the planned administered dose (1000 mg/m^2^) multiplied by the planned treatment occasions (n) according to the protocol.

Dose delays were uncommon. In four patients, nine cycles were delayed mainly due to haematologic toxicities. Dose-reductions were frequently seen both for lenalidomide and gemcitabine (Table 4). In cohort I, 8 out of 13 (62%) treatment cycles were dose-reduced. The corresponding values for cohort II was 44% and for cohort III 71%. Forty-two cycles out of 64 (65.6%) were administered at reduced doses of lenalidomide, gemcitabine or both. Sixteen cycles were administered at a reduced level due to haematologic toxicities (two cycles due to leucopenia grade 2 and one cycle due to leucopenia grade 3; one cycle due to neutropenia grade 1, six cycles due to neutropenia grade 3, five cycles due to neutropenia grade 4 and one cycle due to thrombocytopenia grade 3). Seven cycles were administered at a reduced dose level due to non-haematologic toxicities (one cycle due to urticaria/rash grade 1, three cycles due to urticaria/rash grade 3, one cycle due to neuropathy grade 3, one cycle due to hyperbilirubinemia grade 4 and one cycle due to vomiting grade 2). One cycle was reduced as a consequence of a DLT (see above).

**Table 4:** Dose-reductions by cycle and cohort. Number of cycles with dose-reductions.

**Cohort no.**	**Patients(n)**	**Treatment cycles (n)**	**No. of treatment cycles with dose-reductions (% of total no. of cycles)**	**No. of treatment cycles with dose-reductions of resp. study drug (% of total no. of dose-reduced cycles)**		
				Lenalidomide	Gemcitabine	Both
I	4	13	8 (62)	1 (13)	3 (38)	4 (50)
II	3	9	4 (44)	0 (0)	2 (50)	2 (50)
III	5	42	30 (71)	6 (20)	0 (0)	24 (80)
**Total**	**12**	**64**	**42 (65)**	**7 (17)**	**5/42 (12)**	**30 (71)**

### Safety and toxicity

The most common AEs (all grades) attributed to therapy during the first treatment cycle (Table 5) were hematological and dermatologic toxicities, gastrointestinal (GI) intolerance, and fatigue. Leucopenia and neutropenia were reported in 75% (all grades) of the patients with 78% grade 1–2 and 22% grade 3–4 for leucopenia, and 56% grade 1–2 and 44% grade 3–4 for neutropenia. There was no febrile neutropenia. Thrombocytopenia and anemia was noted in 58% and 25% (all grades), respectively, but only grade 1 or 2. Among non-hematological toxicities, dermatologic toxicities (75% all grades, 50% grade 1 or 2) and GI intolerance were common (67% all grades, but only grade 1 or 2). Nausea was the most common gastrointestinal toxicity (25%), but only grade 1 or 2. Fatigue was common (58%), but all except one episode were grade 1.

**Table 5:** Summary of maximum grade for study related toxicity during treatment cycle number 1.

**Toxicity**	**Number of patients** *					**Total grade 1–4 (% of patients)**
**Grade of AEs** **	0	1	2	3	4	
**Blood/bone marrow**						
Anemia	9	1	2	0	0	3 (25)
Leukopenia	3	3	4	2	0	9 (75)
Neutropenia	3	1	4	3	1	9 (75)
Thrombocytopenia	5	7	0	0	0	7 (58)
Febrile neutropenia	12	0	0	0	0	0 (0)
**Cardiac arrhythmia**						
Supraventricular extrasystoles	11	1	0	0	0	1 (8)
**Cardiac general**						
Left ventricular systolic dysfunction	11	0	0	1	0	1 (8)
Hypotension	11	1	0	0	0	1 (8)
**Constitutional symptoms**						
Fatigue	5	6	0	1	0	7 (58)
Fever, in the absence of neutropenia (ANC <1.0 x 10^9^/L)	11	0	1	0	0	1 (8)
Weight loss	10	2	0	0	0	2 (17)
**Dermatology/skin**						
Urticaria/Rash	9	1	0	2	0	3 (25)
Dry skin	10	2	0	0	0	2 (17)
Pruritus/itching	9	1	1	1	0	3 (25)
Erythema	11	0	1	0	0	1 (8)
**Endocrine**						
Thyroid function	12	0	0	0	0	0 (0)
**Gastrointestinal**						
Constipation	11	1	0	0	0	1 (8)
Diarrhea	10	1	1	0	0	2 (17)
Dry mouth	10	2	0	0	0	2 (17)
Nausea	9	1	2	0	0	3 (25)
Vomiting	11	0	1	0	0	1 (8)
Anorexia	11	1	0	0	0	1 (8)
**Hemorrhage**						
Melena	11	0	1	0	0	1 (8)
**Infection**						
Febrile neutropenia (ANC <1.0 x 10^9^/L, fever ≥38.5°C)	12	0	0	0	0	0 (0)
Viral infection	10	1	1	0	0	2 (17)
**Lymphatics**						
Edema; limb	11	1	0	0	0	1 (8)
**Metabolic**						
ALAT elevated	10	2	0	0	0	2 (17)
ASAT elevated	9	30	0	0	0	3 (25)
Bilirubin elevated	12	0	0	0	0	0 (0)
Creatinine elevated	12	0	0	0	0	0 (0)
**Neurology**						
Dizziness	8	4	0	0	0	4 (33)
Neuropathy—sensory/motor	12	0	0	0	0	0 (0)
**Pain**						
Muscle	10	1	1	0	0	2 (17)
Abdominal	11	0	0	1	0	1 (8)
**Pulmonary/Upper respiratory**						
Dyspnea	10	2	0	0	0	2 (17)
Cough	11	0	1	0	0	1 (8)
Hoarseness	11	1	0	0	0	1 (8)
**Renal**						
Renal failure	12	0	0	0	0	0 (0)
**Vascular**						
Thrombosis/thrombus/embolism	12	0	0	0	0	0 (0)

*Represents the number of subjects (of total n = 12) experiencing adverse event during cycle number 1 with lenalidomide and gemcitabine.

**Graded using NCI CTCAE V 3.0

ALAT = Alanine aminotransferase; ASAT = Aspartate aminotransferase

Grade 3 to 4 adverse events that were possibly related to study treatment for all cycles are listed in Table 6. As noted during and after the first treatment cycle, myelosuppression was common with neutropenia or leucopenia in 83% (all grades) of the patients. 58% and 25% were grade 3–4 neutropenia and leucopenia. In cohort III, all patients that experienced neutropenia (80%) were of grade 3–4 as compared 50% and 33% in cohorts I and II, respectively. Leucopenia grade 3–4 was noted in 40% of the patients in cohort III, but 25% and 0% in cohort I and II, respectively. During the cumulative of treatment cycles, thrombocytopenia was noticed in 75% (all grades, 8% grade 3–4) and anemia was reported 42% (all grades, 8% grade 3–4), without any major difference in frequency and severity between the cohorts. Treatment did not affect lymphocytes, monocytes, eosinophils or basophils counts (data not shown).

**Table 6:** Summary of maximum grade for toxicity (aggregate for all treatment cycles) (NCI CTCAE.V3.0.).

**Toxicity**	**Cohort I (n = 4)**		**Cohort II (n = 3)**		**Cohort III (n = 5)**		**Total (n = 12)**	
	G* 1–4 No** (%)	G 3–4 No (%)	G 1–4 No (%)	G 3–4 No (%)	G 1–4 No (%)	G 3–4 No (%)	G 1–4 No (%)	G 3–4 No (%)
**Blood/bone marrow**								
Anemia	2 (50)	1 (25)	2 (67)	0 (0)	1(20)	0 (0)	5 (42)	1(8)
Leukopenia	3(75)	1 (25)	3 (100)	0 (0)	4(80)	2(40)	10 (83)	3 (25)
Neutropenia	3(75)	2(50)	3(100)	1 (33)	4(80)	4(80)	10(83)	7(58)
Thrombocytopenia	4(100)	1(25)	2(67)	0(0)	3(60)	0(0)	9(75)	1 (8)
Febrile neutropenia	0 (0)	0 (0)	0 (0)	0(0)	0 (0)	0 (0)	0 (0)	0 (0)
**Cardiac arrhythmia**								
Supraventricular extrasystoles	0 (0)	0(0)	1 (33)	0(0)	0(0)	0(0)	1(8)	0(0)
**Cardiac general**								
Left ventricular systolic dysfunction	0(0)	0(0)	0(0)	0(0)	1(20)	1(20)	1(8)	1(8)
Hypotension	1(25)	0(0)	0(0)	0(0)	0(0)	0(0)	1(8)	0(0)
**Constitutional symptoms**								
Fatigue	4(100)	0(0)	3(100)	0(0)	3(60)	0(0)	10(83)	0(0)
Fever, in the absence of neutropenia (ANC <1.0 x 10^9^/L)	0(0)	0(0)	2(67)	0(0)	0(0)	0(0)	2(17)	0(0)
Weight loss	1(25)	0(0)	0(0)	0(0)	2(40)	0(0)	3(25)	0(0)
**Dermatology/skin**								
Urticaria/Rash	2(50)	1(25)	0(0)	0(0)	1(20)	1(20)	3(25)	2(17)
Dry skin	1(25)	0(0)	0(0)	0(0)	2(40)	0(0)	3(25)	0(0)
Pruritus/itching	1(25)	1(25)	0(0)	0(0)	2(40)	0(0)	3(25)	1(8)
Erythema	1(25)	0(0)	0(0)	0(0)	0(0)	0(0)	1(8)	0(0)
**Gastrointestinal**								
Constipation	0(0)	0(0)	0(0)	0(0)	1(20)	0(0)	1(8)	0(0)
Diarrhea	3(75)	0(0)	0(0)	0(0)	2(40)	1(20)	5(42)	1(8)
Dry mouth	1(25)	0(0)	0(0)	0(0)	1(20)	0(0)	2(17)	0(0)
Nausea	0(0)	0(0)	2(67)	0(0)	3(60)	0(0)	5(42)	0(0)
Vomiting	1(25)	0(0)	0(0)	0(0)	1(20)	0(0)	2(17)	0(0)
Anorexia	2(50)	0(0)	1(33)	0(0)	1(20)	0(0)	4(33)	0(0)
Stomatitis	1(25)	0(0)	0(0)	0(0)	0(0)	0(0)	1(8)	0(0)
Perforation	0 (0)	0 (0)	1(33)	1 (33)***	0 (0)	0 (0)	1 (8)	1 (8)
**Hemorrhage**								
Melena	0(0)	0(0)	0(0)	0(0)	1(20)	0(0)	1(8)	0(0)
**Infection**								
Febrile neutropenia (ANC <1.0 x 10^9^/L, fever ≥38.5°C)	0(0)	0(0)	0(0)	0(0)	0(0)	0(0)	0(0)	0(0)
Viral infection	1(25)	0(0)	0(0)	0(0)	2(40)	0(0)	3(25)	0(0)
**Lymphatics**								
Edema; limb	1(25)	0(0)	0(0)	0(0)	1(20)	0(0)	2(17)	0(0)
**Metabolic**								
ALAT elevated	1(25)	0(0)	1(33)	1(33)	3(60)	0(0)	5(42)	1(8)
ASAT elevated	1(25)	0(0)	2(67)	1(33)	3(60)	0(0)	6(50)	1(8)
Bilirubin elevated	1(25)	0(0)	1(33)	1(33)	0(0)	0(0)	2(17)	1(8)
Creatinine elevated	1(25)	0(0)	0(0)	0(0)	1(20)	0(0)	2(17)	0(0)
**Neurology**								
Dizziness	3(75)	0(0)	1(33)	0(0)	1(20)	0(0)	5(42)	0(0)
Neuropathy—sensory/motor	2(50)	1(25)	1(33)	0(0)	2(40)	0(0)	5(42)	1(8)
Mood alteration	0(0)	0(0)	0(0)	0(0)	1(20)	0(0)	1(8)	0(0)
**Pain**								
Muscle	1(25)	0(0)	0(0)	0(0)	1(20)	0(0)	2(17)	0(0)
Abdominal	0(0)	0(0)	1(33)	0(0)	3(60)	1(20)	4(33)	1(8)
**Pulmonary/Upper respiratory**								
Dyspnea	1(25)	0(0)	0(0)	0(0)	1(20)	0(0)	2(17)	0(0)
Cough	1(25)	0(0)	0(0)	0(0)	2(40)	0(0)	3(25)	0(0)
Hoarseness	0(0)	0(0)	0(0)	0(0)	2(40)	0(0)	2(17)	0(0)
**Vascular**								
Thrombosis/thrombus/embolism	0(0)	0(0)	1(33)	1(33)	1(20)	1(20)	2(17)	2(17)

*G = Grade

**Represents the number of subjects experiencing adverse event with lenalidomide and gemcitabine.

*** One patient with grade 4 gastrointestinal perforation underwent acute surgery, died postoperatively day 6 in acute respiratory insufficiency.

The incidence of dermatological toxicity (urticaria/dry skin/pruritus/erythema) during cumulative of treatment cycles was as noted after the first treatment cycle, that is approximately 75% for all skin-toxicities together. Among other non-hematological toxicities, fatigue was the most prominent AE during the whole study period, presented in 83% of the patients, but all episodes were grade 1–2, equally distributed between the cohorts. GI toxicities were common (diarrhea and nausea, both 42%, and anorexia, 33%), but only one episode of diarrhea was grade 3. Among metabolic AEs, elevations of ALAT and ASAT were reported in 42% and 50% (all grades), respectively, but only 8% were grade 3–4 and these occurred after cycle 1 and were not considered DLTs. There was no evidence for any other biochemical toxicities, including thyroid or renal function tests.

Five patients (42%, all of grade 1–2) had neurological side-effects, such as dizziness including light headedness and vertigo. Neuropathy was reported in 42%, mainly as neurosensory toxicity (33%, all grade 1–2) with paraesthesia and sensory alteration. The fourth patient enrolled at dose level 1 (15 mg/day), developed grade 3 neuromotor toxicity with cramps and objective weakness in both hands, interfering with activities of daily living. There was no somnolence reported.

During the entire trial, there were two grade 3 VTEs which occurred after cycle 1. Both patients had unilateral deep vein thrombosis at the end of cycle 2 and 3, respectively, but did not result in treatment discontinuation, but an increment in the prophylactic dose of LMWH. One patient experienced grade 4 gastric perforation 4 days after the last lenalidomide dose in cycle 3. The patient underwent acute surgery but died at day six postoperatively in acute respiratory insufficiency, see section SAEs below.

### Serious adverse events (SAEs)

Four SAEs were reported during the study period out of which one was classified as a DLT (see above) and suspected as a causal relationship to lenalidomide treatment (pat no 135). One patient (pat no 122) experienced a grade 4 gastric perforation 4 days after the last lenalidomide dose in cycle 3. The patient underwent acute surgery but died 6 days postoperatively in acute respiratory insufficiency. At the time of start of treatment, the patient had disease-related pleural effusion that was managed by therapeutic thoracentes. Although a causal relationship with the trial drugs was classified as not suspected it could not be completely ruled out that gastrointestinal perforation was related to study treatment. One patient (pat no 131) required hospital admission for the treatment of grade 3 abdominal pain, and grade 2 vomiting. A relationship with the trial drugs were classified as not suspected, but due to the underlying disease (see section DLT).

During the first week in treatment cycle no 3, 1 patient (pat no 121) developed grade 4 hyperbilirubinemia and grade 2 fever. A causal relationship was classified as not suspected as the patient had an acute pancreatitis due to dysfunction of a biliary stent.

### Prophylactic anti-thrombotic treatment

The prophylactic dose of LMWH was reduced in patient no 111 during treatment cycle no. 1 due to thrombocytopenia grade 1. In patient no. 133, the prophylactic LMWH therapy was interrupted for thirteen days between cycle no. 12 and 13, due to thrombocytopenia grade 2. In one other patient (no. 134), LMWH therapy was withheld for three days during cycle no. 1 due to melena grade 1 that was not considered to be related to therapy but to underlying disease.

### Secondary primary malignancies (SPMs)

There were no SPMs.

## Discussion

This is the first phase I dose escalation trial report combining lenalidomide and standard chemotherapy with gemcitabine in patients with advanced adenocarcinoma of the pancreas. Detailed toxicity data is crucial to recommend a safe and tolerable dose of lenalidomide in combination with gemcitabine. Based on the results from this study, the MTD of lenalidomide administered daily on days 1–21 of a 28 day cycle was defined as 25 mg per day in combination with gemcitabine 1000 mg/m^2^ on days 1, 8 and 15 as first line treatment in patients with advanced pancreatic cancer. This dose level is recommended for further studies in the phase II setting.

DLT consisted of one episode with symptomatic, but transient, grade 3 heart failure, at the highest dose level of lenalidomide (25 mg/day) in combination with standard dosing of gemcitabine. According to the Summary of Product Characteristics (SPC) of lenalidomide, congestive heart failure was listed as a common grade 3/4 AE (in 1–10% of patients with MM) [13]. In phase I trials in solid tumors, when using lenalidomide as monotherapy [23, 24] or in combination with chemotherapy [25, 27], no events of cardiac failure has been reported, but episodes of palpitations, arrhythmias and increments in QTs interval have been noted.

The most common lenalidomide-related adverse events are myelosuppression with neutropenia/leucopenia and thrombocytopenia, VTEs, fatigue and skin-toxicity [14, 38]. All those side-effects were also frequently seen in the present study. Although neutropenia was the most common grade 3–4 toxicity (58%) in the present study, there was no febrile neutropenia. This is in line with one study [26, 27] but in contrast to results achieved in other phase I/II trials combining lenalidomide and lenalidomide-like compounds with gemcitabine [26, 39], or other cytotoxic drugs [25], where episodes of neutropenia frequently have been associated with fever and usually been classified as DLT. An explanation for this discrepancy might be the limited number of patients in our study and differences in the dose escalation schedules. Compared to treatment with lenalidomide as single agent in solid tumors [23, 24], the rate of neutropenia, leucopenia and thrombocytopenia was increased in all cohorts with the addition of gemcitabine. Furthermore, as in the previous studies [23, 24], neutropenia and leucopenia seemed to be dose dependent and reversible. Thrombocytopenia was mild with only one grade 3 and no grade 4 events as compared to 20% (grade 3 + grade 4) observed in an earlier study with the same combination [26, 27]. The rate of thrombocytopenia was however increased compared to lenalidomide as a single agent [5, 40], or with gemcitabine combined with other immunomodulating agents [26, 39].

Compared with other malignancies, patients with advanced pancreatic cancer have an increased risk for venous thromboembolism (VTE) [41, 42]. Chemotherapy per see also increases the risk of VTE in pancreatic cancer patients [43]. Furthermore, VTE is a known complication of lenalidomide with concurrent administration of high-dose dexamethasone or chemotherapy in MM [44], but also in patients with advanced pancreatic cancer [45]. The risk of VTE toxicity was of special concern also in our study, why all patients were treated with LMWH. The rate of observed grade 3 VTEs in this trial of 17% is within the same range as reported for other studies using lenalidomide or pomalidomide in combination with gemcitabine for advanced pancreatic cancer, using aspirin as prophylactic anticoagulant [26, 27, 45]. As in concert with other trials, no significant added toxicity was observed from the addition of a prophylactic LMWH schedule to chemotherapy [46]. No patients discontinued protocol therapy due to VTEs. There were only a few episodes of LMWH interruptions or dose-reductions (all due to thrombocytopenia), indicating that the addition of LMWH is safe.

Urticaria/rash, dry skin and mouth and pruritus are the most common dermatological toxicities reported for lenalidomide [38]. The episodes of those AEs in the present study were noted in 9 out of 12 (75%) patients with 25% of grade 3 but no grade 4. One patient (no. 113) developed concomitant grade 3 urticaria and grade 2 erythema during the first treatment cycle and the lenalidomide administration was temporarily held. Interestingly, all dermatological toxicities except for one were noted during treatment cycle number 1. However, the high frequency of dermatological toxicity observed in the current trial in combination with gemcitabine, compared to lenalidomide as single-agent in solid tumors [23, 24], or in combination with dexamethasone in MM [14], might be attributed to overlapping toxicity.

One patient in cohort II experienced grade 4 gastrointestinal perforation after the third treatment cycle and underwent acute surgery. Gastrointestinal perforation is not described in the formal European product information for MM [13], but described in the attached report on GI Safety citing the US product information that includes data from patients with myelodysplastic syndromes [47]. A risk of gastrointestinal perforation with antiangiogenesis inhibitors, such as bevacizumab [48], including pomalidomide combined with gemcitabine [26], mandates close attention to this issue in future studies.

The risk of SPM must be considered in lenalidomide-treated patients. The increased risk of SPMs has mainly been related to the use of lenalidomide maintenance therapy in MM [49], with reported median time to the diagnosis of SPMs of 28–30 months [50]. In the current study with relatively few patients and a short survival we did not detect any SPM.

The MTD dose of lenalidomide in this study is basically the same as recommendations in other phase I studies [22–25, 35], with respect to some specific differences in days “off” and “on”-administration of lenalidomide. If this schedule is proven to be effective in future studies we believe that there will be no strong barriers to implementation. However, the patients were in a good performance status at baseline limiting the generalizability of our findings. Of note is that the recent study by Infante et al previously referred to in this article suggests that lenalidomide + gemcitabine is not an effective treatment due to toxicity and lack of antitumoral effects. This study started in parallel with our study, it is american and it used a different design compared to ours **[27]**.

In conclusion, we show that the combination of lenalidomide and gemcitabine is tolerable and safe as first line treatment in patients with advanced pancreatic cancer. The tolerability profile demonstrated in the dose escalation schedule of lenalidomide suggests that the optimal dosing of lenalidomide is 25 mg daily on days 1–21 and of gemcitabine 1000 mg/m^2^ on days 1, 8 and 15 of a 28 day cycle. This dose level of lenalidomide was further evaluated in a phase II trial with the aim to analyse the immunological response and clinical efficacy of lenalidomide on combination with gemcitabine as first line therapy in patients with advanced pancreatic cancer.

## Supporting Information

S1 Flow Diagram (PDF)

S1 Protocol (PDF)

S1 TREND Checklist Trend Checklist. (PDF)
