# Supplementary figures and images for: A Phase I Dose-Escalation Study of Lenalidomide in Combination with Gemcitabine in Patients with Advanced Pancreatic Cancer
Source: PLoS One. 2015 Apr 2;10(4):e0121197. doi: 10.1371/journal.pone.0121197 (PMC4383423; doi:10.1371/journal.pone.0121197)

## CONSORT 2010 Flow Diagram

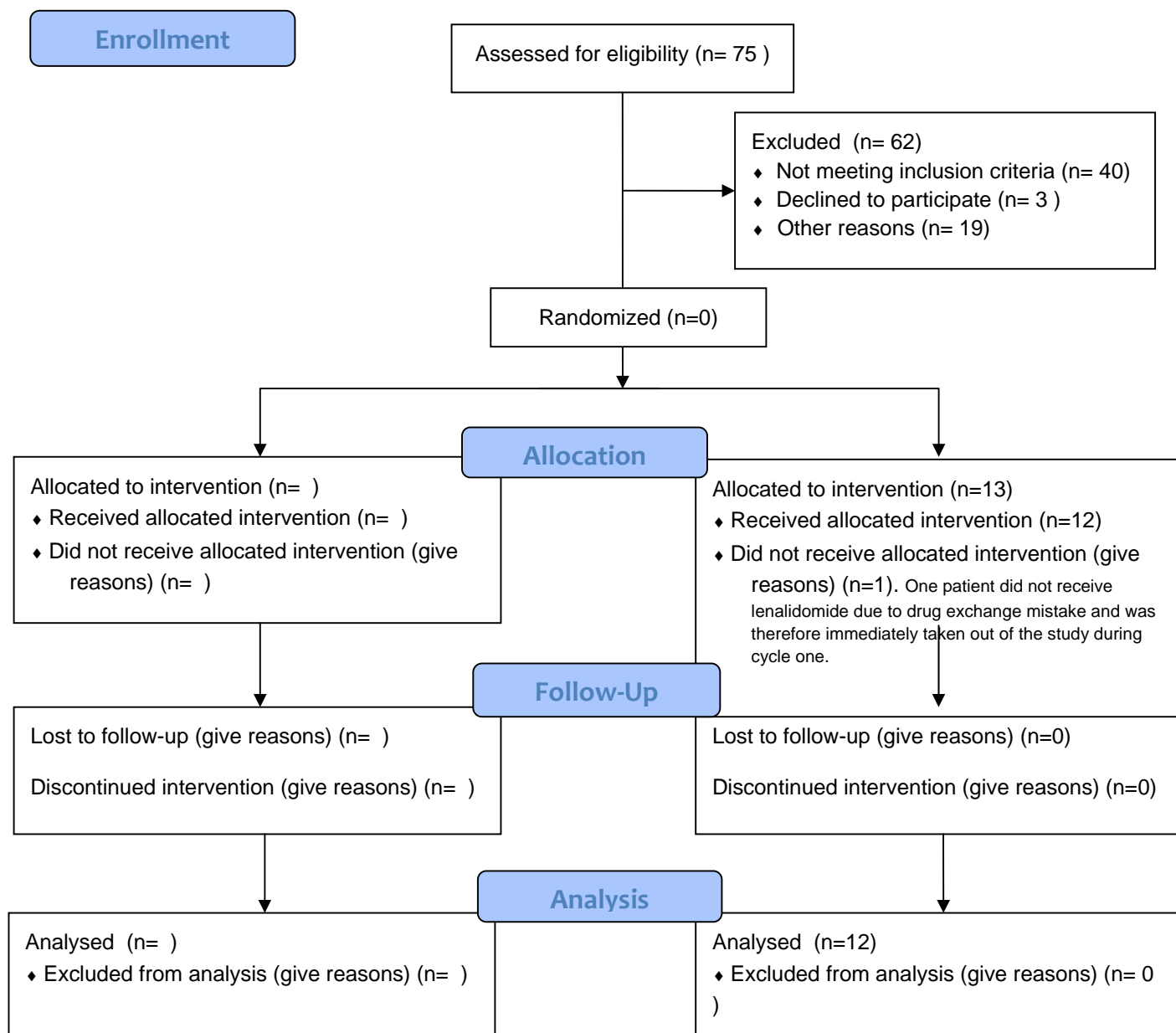

Supplement: S1 Flow Diagram — (PDF) [file pone.0121197.s001.pdf]
